# Two Cases Uterine Hydatids

**Published:** 1845-05

**Authors:** Jno. Evans

**Affiliations:** Attica, Indiana


ILLINOIS

MEDICAL & SURGICAL JOURNAL.

VOL. II. MAY, 1845. NO. 2.

*Two Cases Uterine Hydatids.* By JNO. EVANS, M. D., of Attica, Indiana.

Uterine Hydatids seem to have attracted attention as early as the fifth century, as an allusion to them is found in the writings of Ætius; since which time numerous authors have successively given a more brief or extended description of the disease. But until Madam Boivin wrote, no full or systematic account was given of it. And since the publication of her memoir on the subject, in 1827, however erroneous some of her opinions may prove to be, authors seem to have contented themselves with her statements, and to have concluded that there is little else to do, than "to verify and strengthen her inferences." Even Prof. Gross, in his great work on Pathological Anatomy, has adopted, without dissent, her opinions.

As we are yet in the dark in reference to the cause, mode of development, and, to a great extent, the nature and proper treatment of the disease; the following cases are reported with a hope that they may add something to the amount of facts already gathered on the subject. They are interesting in their bearing on the subject of the origin of Hydatids, as it is a question of much forensic importance.

I am led to believe that Uterine Hydatids are much more common than the student of Dewees would suppose; as that veteran, in the course of a long life and a more extensive practice in Midwifery, than has fallen to the lot of any other American, found but a single case of the disease. I have had an account of several cases, falling under the observation of professional acquaintances, in addition to those here reported. And when we remember the amount of Obstetrical practice which is attended to by those who are too ignorant, or too careless in their observations to notice a case of the kind; we may consistently conclude that many cases of Uterine Hydatids exists without being detected at all.

*Case 1st.*—Mrs. B., aged 25 years, of lymphatic temperament, light complexion and light hair; who had two healthy children, and enjoyed tolerable good health until the summer and fall of 1842, when she suffered from repeated attacks of intermittent fever; observed a gradual tumefaction of the abdomen, which led to the conclusion that she was pregnant.

At a period which she supposed to be the fifth or sixth month of gestation, she was seized with labor, and I was called to treat the threatened miscarriage. Before I arrived there had been expelled from the uterus a quantity of semi-transparent gelatinous fluid, which on cooling became more tenaceous, until it was of the consistency of the white of an egg, which was completely filled with hydatids of all intermediate sizes, between that of a grain of wheat and a hazel nut, and numbering many hundreds. I preserved for office inspection, a quart, which was about half the quantity discharged. There were no membranes observed.

The labor speedily subsided and was attended, at the time, with but little hæmorrhage or other unpleasant symptoms, and she was soon able to sit up most of her time, but did not entirely recover, owing to a torpid condition of the liver, an atonic condition of the stomach, and an occasional attack of the uterine hæmorrhage, which last, was generally arrested by a decoction of Ergot, or the use of sugar of lead and Dover's powder.

Chologues, alteratives and tonics were used, as they seemed to be indicated for six months, without any permanent advantage, and the system gradually gave way. The attacks of uterine hæmorrhage became more frequent, more profuse and more difficult to control, until anemia and general anasaica were induced. This decline was attended with almost constant pain in the iliac region, and in the regio-pubis, without a corresponding tenderness, no doubt produced by the hydatids in the ovaries and uterus. Early in June, 1843, the flooding became profuse, and she sunk into a deep coma, with insensibility, the breathing became sterterous, and she died.

*Autopsy 20 hours after death.*—Brain presented a healthy appearance, except that, contrary to the apopletic symptoms of which she died, it partook of the general anemia, a case in illustration of anemic apoplexy. The thoracic and abdominal viscera presented nothing worthy of remark until the ovaries were examined, which were enlarged to the size of a quail's egg, and on being divided were found full of hydatids of various sizes. The frimbriated extremities of the Fallopian tubes were highly injected with blood, and of a bright florid color.

The uterus was slightly enlarged, but presented a normal appearance externally. Within its cavity, near the middle of the posterior wall, there was a regular tumor of the size of a peach-stone, from the centre of which a polypus of gelatinous variety, about the size of a pea, was suspended by a narrow foot-stalk. Upon dividing the tumor it was found to contain a number of small hydatids, with two or three bodies containing all the characteristics of the above described polypus. On making further division of the posterior parieties of the uterus, there were found embeded in the substance, four or five isolated hydatids, which, like those expelled by labor, those found in the ovaries, and those of the tumor, presented all the appearances of the genuine acephalocyst.

This is, perhaps, the first case reported of a polypus being attached to the side of the uterus. The hæmorrhage, which was the cause of the death was not attributable to the hydatids, but to polypus. The irritation produced by the hydatids may have caused a greater determination to the uterus, "*ubi irritatio ibi affluxit*," and this have increased the bleeding of polypus.

*Case 2nd.*—Mrs. B., æt. 55 years, of robust constitution, bilious temperament, dark complexion and dark hair; of German extraction and a native of Pennsylvania,—had raised a family of healthy children, the youngest of whom was twelve years old, when she observed a gradual tumefaction of the abdomen, which excited suspicion that she had become pregnant in her old age. At the usual age, she had undergone "the change of life," and for a number of years had no sign of the catamenial flux, during which period she had enjoyed good health.

On the 1st of August, 1841, I found her in moderate labor, with slight hæmorrhage. Prescribed a free use of acet. Plumbi and Pulv. Doveri, which arrested both.

August 2d. Labor returned; and when I arrived, she had been delivered of a mass of hydatids, in a jelly like substance of about the consistence of the coagulum of blood. I attempted to preserve a quantity, as a specimen, but did not succeed. For two days, small quantities of this matter were discharged at intervals; there was but little flooding, and she speedily recovered.

About a year afterward, she informed me that she had enjoyed good health, and regularly menstruated since the hydatids were discharged.

We find Valesneri, Desormeaux, Mad. Boivin, Prof. Gross, and others, concurring in the opinion that uterine hydatids are always the product of impregnation, and are a degeneration of the placentæ, ovum or membranes, while Percy saw fit to acquit "a young religieuse" of a charge of incontinence, by declaring, that "vesicular moles are merely hydatids." And the only case Dewees found, was in a widow lady, of good character, whose husband had been dead three years. But here we have one case in which hydatids are found in the texture of the uterus, forming a tumor within its cavity, while a copious crop were formed rapidly and discharged without any sign of ovum or membranes. And another occurring at an age which is almost always beyond the period of susceptibility to impregnation.

These cases are sufficiently strong in opposition to the theory of hydatids of the uterus being the result of impregnation, to justify a jury in leaning to the side of mercy. For although the impregnated ovum is sometimes the seat of hydatids, as Dr. Atlee's case, and many others clearly prove, that fact is not quite broad enough to justify the conclusion, that they are only found in connexion with it.

Attica, Ia., March 10, 1845.

